# Functional Profiles of Phyllosphere and Rhizosphere Metagenomes Differ Across Milkweed Species

**DOI:** 10.1111/1758-2229.70150

**Published:** 2025-07-15

**Authors:** Thorsten E. Hansen, Laramy S. Enders

**Affiliations:** ^1^ Tropical Pest Genetics and Molecular Biology Research Unit, Daniel K. Inouye US Pacific Basin Agricultural Research Center, Agricultural Research Service US Department of Agriculture Hilo Hawaii USA; ^2^ Department of Entomology Purdue University West Lafayette Indiana USA

**Keywords:** functional profiling, metagenomics, milkweed species (*Asclepias* spp.), monarch butterfly (*Danaus plexippus*), phyllosphere, plant secondary metabolites (PSMs), rhizosphere

## Abstract

Monarchs and their milkweed hosts are well studied for mechanisms of plant defence and insect counter‐defence, but little is known about their associated microbiomes in this iconic system. It is unclear how herbivore‐induced changes in milkweed microbiomes are linked to chemical defensive responses (i.e., plant secondary metabolites). Metagenomics was used to characterise functional gene profiles of bacterial communities associated with plant tissues and monarch caterpillars feeding in milkweed spp. ( *Asclepias curassavica* L., 1753, *Asclepias syriaca* L., 1762, and *Asclepias tuberosa* subsp. *tuberosa*). We compared the influence of monarch feeding and milkweed spp. on phyllosphere and rhizosphere communities. Shotgun metagenomic sequencing was used to profile microbial diversity, predict annotative functions, assess microbial plant secondary metabolite (PSM) degradation potential, and look for enrichment of PSM degradation pathways. Findings show phyllosphere and rhizosphere microbiomes have distinct functional gene profiles and the presence of potential PSM metabolism genes that varied across milkweed spp. Rhizosphere metagenomes had more genes and metabolic pathways involved in degradation/detoxification of known classes of PSMs. In relation to milkweed defensive chemical profiles, we saw an inverse pattern of PSM metabolism pathways and enzymes. Overall, results suggested greater potential for PSM metabolism in rhizosphere compared to phyllosphere communities.

## Graphical abstract

We characterised milkweed microbiomes using a metagenomics approach to investigate the functional potential of endophytic phyllosphere and rhizosphere communities. We found evidence that the composition of potential PSM genes varied across milkweeds in spp. specific responses, for both phyllospheres and rhizospheres, and in response to monarch feeding.

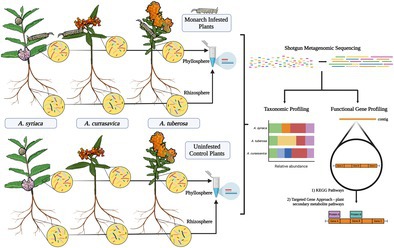

## 1 Introduction

Microbial communities are essential to plant health and physiological function (Bhadrecha et al. 2023; Chauhan et al. 2022; Gong and Xin 2021; Singh et al. 2023; Sohrabi et al. 2023; Xu et al. 2022). The ecological interactions between plant‐associated microbiomes and their hosts are complex. A myriad of abiotic and biotic factors directly and indirectly influences microbiota compositions associated with plant tissues, including phyllospheres (leaves) and rhizospheres (roots) (Gong and Xin 2021; Kumar et al. 2023; Munoz‐Ucros et al. 2021; Park et al. 2023; Sharma et al. 2023; Xu et al. 2022). Host plant immunity and defences are hypothesised to impose direct selective pressure on plant‐associated microbiomes (Fitzpatrick et al. 2020). For example, production of reactive oxygen spp. (Jones and Smirnoff 2005) and callose deposition in tissues can prevent microbial invasion (Nishad et al. 2020; Pršić and Ongena 2020), whereas volatile compounds and plant secondary metabolites (PSM) can attract or repel a range of microbes (Hammerbacher et al. 2019; Jacoby et al. 2021). Additionally, plants must contend with herbivorous insects that damage tissues and deplete vital nutrients. Insect herbivore feeding not only triggers defences like PSM production (Karban and Baldwin 2007) but can induce changes in rhizosphere and phyllosphere microbial communities (French et al. 2021; Humphrey and Whiteman 2020; Tronson et al. 2022). However, it is unclear how herbivore‐induced changes in plant microbiomes are linked to chemical defensive responses and production of PSMs.

A growing number of studies predict plant defences play a key role in shaping microbiome composition across plant spp. Recent research shows systematic expression of PSMs in plant roots changes depending on surrounding soil microbiome (Korenblum et al. 2020). However, PSMs may also directly or indirectly impact rhizosphere microbiome composition. One possibility is PSMs are a carbon source, and some microbial taxa can metabolise these compounds. Additionally, PSMs may negatively impact specific taxa through direct antimicrobial effects. Research shows PSMs from several different chemical groups (e.g., coumarins, benzoxazinoids, camalexin, glucosinolates, triterpenes) have antimicrobial effects that in turn could alter microbial community assembly and composition (Jacoby et al. 2021; Pang et al. 2021). For example, several studies have identified microbial genes that encode for enzymes predicted to metabolise aromatic compounds (Babalola et al. 2020; Wicaksono et al. 2021); and aromatic, phenolic compounds are known to exhibit antimicrobial properties (Kumar et al. 2020). The high diversity of PSMs and existence of slow‐growing microbes adapted to specialised metabolic niches (Zhalnina et al. 2018) suggest expression of PSMs in roots and leaves could impact microbiome assembly more broadly across plant tissues. However, the impact of PSM production on rhizosphere or phyllosphere community assembly is unclear. Studies are needed that investigate relationships between herbivore‐induced defence responses and changes in plant‐associated microbiomes.

PSMs are found across most plant spp., but the diversity and abundances of these compounds vary (Kessler and Kalske 2018; Moore et al. 2014). For example, phylogenetically distinct compounds exist, such as cardenolides in *Asclepias* spp. (milkweeds) (Agrawal et al. 2012), making milkweed an interesting non‐model system to explore interactions between plant defences and associated microbial communities. Milkweed spp. are well known for producing toxic cardenolides, but there is considerable variation in expression levels and overall anti‐herbivore defensive profiles in both field and glasshouse plants (Rasmann and Agrawal 2011; Züst et al. 2019). For example, tropical milkweed ( *Asclepias curassavica* ), common milkweed ( *Asclepias syriaca* ), and butterfly weed ( *Asclepias tuberosa* ) exhibit a spectrum of chemical and physical defences. Tropical milkweeds are known to have high concentrations and diversity of cardenolides, with common milkweed having a smaller range of concentrations and diversity, whereas cardenolide levels in butterfly weed are negligible (Agrawal and Fishbein 2006, 2008; Rasmann and Agrawal 2011). Most milkweeds can excrete latex from their tissues as both a physical and chemical defence, as latex transports cardenolides throughout tissues (Agrawal et al. 2012). Common milkweed produces high levels of latex, tropical milkweed is a moderate latex producer, whereas butterfly weed makes miniscule amounts (Agrawal 2004; Agrawal et al. 2014; Agrawal and Fishbein 2006). These three spp. also have distinct mechanical defence traits.

For example, common milkweed has high trichome densities compared to the other two spp. (Agrawal 2004; Agrawal and Fishbein 2006, 2008; Fishbein et al. 2018) but butterfly weed's trichomes are rigid and inconsistent in length (likely providing better protection) (Colvin et al. 2013). Butterfly weed has tougher leaves, followed by tropical milkweed, and lastly common milkweed (Agrawal 2004; Agrawal and Fishbein 2006). Altogether, defensive traits of milkweeds potentially make for a unique microbial ecosystem to compare how plant‐associated microbiomes responded and evolved to PSMs.

Recent work found distinct microbial communities associated with phyllospheres and rhizospheres of two milkweed spp., *A. curassavica* and *A. syriaca* (Hansen and Enders 2022), but it is unclear to what extent these differences are linked to host plant PSM production. Cardenolides are systemically produced in response to microbial infection and herbivore feeding damage throughout milkweed tissues (Agrawal et al. 2012), which suggests microbes are likely exposed to PSM to varying degrees. The endophytic proportion of the phyllosphere may be more directly affected by milkweed PSMs produced in leaf tissues, compared to the rhizosphere where PSMs would need to be exudated outside the root. Further, while some cardenolides exhibit antimicrobial activity (Akhtar et al. 1992; Bertol et al. 2011; Jacobsohn and Jacobsohn 1985), direct antibiotic effects are unlikely for microbes that lack Na^+^/^+^K‐ATPase enzymes specifically targeted by cardenolides (Agrawal et al. 2012). Alternatively, cardenolides could act as a carbon source for microbes with necessary metabolic capabilities. Although cardenolide metabolism has not been experimentally shown in milkweed‐associated microbiomes, the human gut microbe *Eggertella lenta* encodes for gene Cgr2, a putative cardiac glycoside reductase. *E. lenta* can inactivate digoxin, among several other cardiac glycosides, but does not appear to use byproducts for growth (Koppel et al. 2018). Milkweeds express additional PSMs including alkaloids, flavonoids, lignans, pentacyclic triterpenes, phenolics, saponins, steroids, and tannins (Agrawal et al. 2009, 2012; Araya et al. 2012; de Leão et al. 2020; Jawale 2014). Thus, a wide range of potential PSMs could impose selective pressures on associated microbial communities through several mechanisms. However, the functional potential of milkweed microbiomes is currently unexplored, and it is unclear if root and leaf‐associated microbes can metabolise milkweed defensive chemicals.

The goal of this study is to investigate how rhizosphere and phyllosphere microbiomes functionally differ across milkweed spp. known to vary in defensive chemical profiles. Specifically, we addressed the following: (1) Is there evidence of microbial PSM metabolism and is it distinct across milkweed spp.? (2) If PSM metabolism is found, is it more prevalent in rhizosphere or phyllosphere communities? (3) Does insect herbivore feeding cause changes in potential microbial PSM metabolism? To address these questions, we used shotgun metagenomic sequencing to characterise the functional gene profiles and identify bacterial metagenome assembled genomes (MAGs) associated with both the plant phyllosphere and rhizosphere across three milkweed spp.: tropical milkweed ( *A. curassavica* ), common milkweed ( *A. syriaca* ), and butterfly weed ( *A. tuberosa* ). We hypothesised defensive chemical profiles impose positive directional selection on plant‐associated microbiomes causing an enrichment of detoxification genes across bacterial genomes. We took a targeted gene approach that focused on characterising evidence of PSM metabolism in bacterial metagenomes, specifically associated with cardenolides, saponins, phenolics, alkaloids, tannins, and flavonoids. Specifically, we predict enrichment of PSM metabolism genes is greatest in metagenomes of (i) plant spp. with higher expression/concentrations of cardenolides (i.e., tropical and common milkweed), (ii) phyllosphere compared to rhizosphere microbiomes, and (iii) in plants exposed to monarch caterpillar feeding compared to un‐infested plants.

## 2 Experimental Procedures

### 2.1 Plant and Insect Materials

The three milkweed spp. used in this study were: tropical milkweed ( *Asclepias curassavica* L.), common milkweed ( *Asclepias syriaca* L.), and butterfly milkweed ( *Asclepias tuberosa* L.). Seeds were purchased from Everwilde Farm Inc. Before germination and planting, *A. syriaca* and *A. tuberosa* seeds were cold stratified at 4°C on moist filter paper for 2–3 weeks, surface sterilised with 5% bleach, germinated at room temperature (21°C ± 1°C) inside a growth chamber, and planted in autoclaved potting soil (150 g/pot). Monarch eggs were obtained from Shady Oak Butterfly Farm Inc. Monarch eggs were kept at room temperature (21°C ± 1°C) inside a growth chamber. Once larvae hatched, they were fed on *A. syriaca* leaves until they reached 2nd instar and were used in the experiment described below.

Seedlings were grown in glasshouse conditions (Photoperiod 14 h: 10 h, light: dark, 26°C day: 20°C night) for 86 days. Pots were watered every 6 h for 10 min, starting at 10:00 PM with drip irrigation stakes. All plants were fertilised every 2 weeks with a fertiliser solution from the bottom to prevent introduction of minerals that could influence soil microbial composition and minimise disturbance. Each plant was enclosed with a custom‐made cage (3.8 L (litre)—14.3 cm length × 12.7 cm width × 25.7 cm height) from the start of plant growth till the end of the experiment.

### 2.2 Starting Soil Microbiomes

All plants were inoculated with custom soil microbiome slurry when seeds were planted. The inoculum was applied to the top of the soil. To make the soil microbiome slurry, soil samples were collected at three separate Indiana (USA) field sites where common milkweed plants were present (Table S1).

Before preparing the soil microbiome slurry all glassware and tools were autoclaved for sterilisation and all pots (10 cm diameter) soaked in 5% bleach solution and washed with deionised water. Soil samples were combined with sterile Milli‐Q ‘ultrapure’ water in a 1:1 ratio to create the inoculation slurry. The soil slurry was mixed on a shaker at 200 rpm for 30 min before use. After mixing on the shaker, 15 mL of inoculum was applied per pot at 0.1 g mL^−1^ (achieving a 10% inoculum).

### 2.3 Experimental Design and Sample Collection

In total, 120 plants were grown (40 plants/spp.), half of which were infested with monarch caterpillars (a single 2nd instar larvae/plant) and the other half had no monarch caterpillars (un‐infested controls—never encountered a monarch during experiment). Comparison of infested plants to un‐infested control plants allows for identification of insect feeding induced functional differences in phyllosphere and rhizosphere microbiomes, including whether there are milkweed species specific patterns and/or general functional changes shared across milkweed species. Plants were infested at 86 days, and monarch larvae were allowed to feed for 4 days. The infestation period (4 days) was chosen based on (1) previous research showing monarch herbivory causes significant induction of plant defences (e.g., cardenolide production) that peaks 2–3 days after feeding and can remain elevated for 10 days (Agrawal et al. 2012) and (2) preliminary feeding assays used to determine an optimal feeding duration that would inflict damage to the plant to induce defences but avoid complete defoliation of the plant (i.e., leave tissue for analysis). Samples were collected for analysis of rhizosphere and endophytic phyllosphere microbial communities following methodology developed in Hansen and Enders (2022), except DNA extractions were done with the ZymoBIOMICS DNA/RNA Miniprep Kit (Zymo Research, Tustin, CA).

### 2.4 Metagenomic Sequencing and Construction of Metagenomic Contigs

DNA samples were randomly subsampled across replicate plants per milkweed spp. to achieve equal distribution between plant spp., monarch infestation and microbiome source (roots or leaves). We included five DNA samples per treatment combination for metagenomic sequencing (75 total DNA samples). All DNA samples were sent to the University of Minnesota Genomics Center (1475 Gortner Ave. 28 Snyder Hall, MN) for whole shotgun metagenomic sequencing. After quality control, libraries were created using the Illumina Nextera XT DNA kit and sequencing (150 bp paired end reads) was performed on an Illumina NovaSeq 6000 with an S4 flow cell. Short‐read metagenomic sequencing was performed, as it is the current standard for successfully capturing the genomic sequences of communities of microbes and has been used for microbiome research in human systems (Duru et al. 2024; Kim et al. 2021; Troci et al. 2023), insects (Ettinger et al. 2022; Gallardo et al. 2024; Meng et al. 2022; Mies et al. 2024), and plants (Huang et al. 2024; Su et al. 2022; Wu et al. 2022).

All initial sample demultiplexing was done by the University of Minnesota Genomics Center with Illumina software. Read quality assessment was conducted for each individual paired read file using FastQC (v 0.11.9) (FastQC, 2016) and Trimmomatic (v 0.39) was used to remove low quality reads, Illumina Nextera adapters, and trim reads across samples (Bolger et al. 2014). Total read quality was assessed across paired read files using MultiQC (v 1.11) (Ewels et al. 2016). To control for lab contamination, four blank DNA extractions were included (one per each extraction kit) and used to create a negative control blank database. To filter for potential DNA contamination, we used the program KneadData (v 0.10.0) (https://bitbucket.org/biobakery/kneaddata), with a human genome and *A. syriaca* genome reference databases, aligning our paired end reads and removing contaminant sequences, thus producing cleaned paired end reads.

High quality paired end reads, for rhizosphere and phyllosphere, and singleton phyllosphere reads were co‐assembled into metagenomic data sets using MEGAHIT (v 1.2.9) (Li et al. 2015). Assemblies were quality checked using the program QUAST (v 5.2.0) (Gurevich et al. 2013). A complete summary of sequencing results can be found in Table S2.

### 2.5 Taxonomic Profiling of Phyllosphere and Rhizosphere Communities

To characterise microbial community structure and diversity across plant spp. and herbivore treatments we used the programs Kraken2 (v 2.1.2) (Wood et al. 2019) and Bracken (v 2.7) (Lu et al. 2017) for taxonomic identification. A bacterial Kraken2 database was built and used with paired‐end reads. Kraken2 is commonly used for taxonomic classification of metagenomic datasets (Edwin et al. 2024; Héctor et al. 2024; Mannion et al. 2023). Kraken2 uses a taxonomic identification system that differs from standard metabarcoding approaches because it maps reads to known genomes rather than short sequences from specific genes of interest. Specifically, it uses exact k‐mer matches to query genomic database sequences for the lowest common ancestor of all genomes containing the k‐mer. In this study, OTUs were assigned by matches to nucleotide sequences from the RefSeq genomic database (v 205) (O'Leary et al. 2016) containing selected assembled genomes available in GenBank. In our case, as well as other metagenomics studies, taxonomic assignment to ASV/strain level is not possible as much larger and diverse communities will share more related genomic information (Snipen et al. 2021; Yang et al. 2019) and although 16S rRNA genes are sequenced, coverage and depth may limit their distribution across organisms (Quince et al. 2019; Shah et al. 2011).

For alpha diversity, read counts were normalised by rarefying to even sampling depths across samples. For beta diversity, Cumulative Sum Scaling (CSS) was used to normalise read counts through the package metagenomeSeq (v 1.30.0) (Paulson et al. 2013). This normalisation technique has been previously used when comparing microbiomes between soil, leaves, and insects (Hannula et al. 2019; Hansen and Enders 2022).

We compared standard alpha and beta diversity metrics using the phyloseq (v 1.32.0) (McMurdie and Holmes 2013), vegan (v 2.5.7) (Oksanen et al. 2022), and rstatix (0.6.0) (Kassambara 2023) packages in R. To compare spp. richness and evenness, we used a Kruskal Wallis rank sum test. Separate tests were run to compare plant spp. and insect presence for each alpha diversity metric. Differences in bacterial community structure were assessed through PERMANOVA of beta diversity (Bray‐Curtis) and visualised using Principal Coordinates Analysis (PCoA). The PERMANOVA model for rhizosphere and phyllosphere microbiomes included the following: plant species, insect presence, and plant species × insect presence. Dispersion across samples was also tested using PERMDISP and pairwise comparisons we performed for plant spp. (Martinez Arbizu 2020).

### 2.6 Functional Gene Profiling of Phyllosphere and Rhizosphere Microbiomes

To predict open reading frames of potential genes across our contigs, the program Prodigal (v 2.6.3) was used (Hyatt et al. 2010). Subsequently, all predicted protein‐coding genes via Prodigal were clustered into a gene catalogue using CD‐HIT (v 4.8.1) at 90% nucleotide identity and 90% coverage (Fu et al. 2012). The open reading frames were then annotated with a combined approach where sequences were searched against the eggNOG database (v 5.0.2) (Huerta‐Cepas et al. 2019) via BLASTX through DIAMOND (v 2.0.15) (Buchfink et al. 2021) using eggNOG mapper (v 2.1.7) (Cantalapiedra et al. 2021).

We identified predicted molecular functions of bacterial genes using KEGG Orthology (KO) annotation information and associated KEGG pathways. Indexes of gene catalogues were created using BWA (Li 2013). We then mapped reads to gene catalogues using BWA‐MEM (v 0.7.17) (Li 2013) and Samtools (v 1.1.17q) (Li et al. 2009). Reads mapped to genes with KO information were used to calculate the abundance of predicted genes within functional categories.

Differences in functional gene profiles were examined using PERMANOVA of Bray–Curtis dissimilarity calculated from gene counts of annotated bacterial genes with assigned KO terms, and visualised using Principal Coordinates Analysis (PCoA). Separate analyses were performed for rhizosphere and phyllosphere communities. Comparisons were made across plant species and herbivore treatments using the following PERMANOVA model: plant species, insect presence, and plant species × insect presence. Dispersion across samples was also tested using PERMDISP. Additionally, differential gene abundance was performed using the package edgeR (v 3.42.4) (Robinson et al. 2010). Differentially abundant genes with log2 fold change of −2 ≤ or ≥ 2 and Padjusted values ≤ 0.05 were identified as enriched or reduced (i.e., differentially abundant). To visualise functional profiling results, we grouped differentially abundant genes by KEGG functional categories (level two) based on KEGG pathway assignments. Genes mapped to multiple KEGG pathways were counted in each KEGG functional category to which they were assigned. Overrepresentation analysis (ORA) identifies enriched pathways per gene set using Fisher's exact test for significance. ORA was performed for differentially abundant genes, between each treatment comparison, using clusterProfiler (v 4.10.1) (Wu et al. 2021).

A separate analysis was conducted using our custom PSM detoxification database. First, we searched keywords against the UniRef90 protein database (v 1.0) (Suzek et al. 2015), filtered by Bacteria (eubacteria): (‘demethylase’ and ‘xanthine oxidase’) for alkaloids, (‘cytochrome’, ‘glucohydrolase’, ‘glycoside hydrolase’, ‘digoxin reductase’, ‘cytochrome c‐type protein cgr1’) for cardenolides, (‘phloretin hydrolase’, and ‘enoate reductase’) for flavonoids, ‘phenolic’, ‘phenolics’, ‘saponin’, and ‘tannin.’ All the downloaded FASTA sequences were joined into a single FASTA file, which gave a total database of potential 1,152,814 microbial PSM genes. EggNOG mapper (v 2.1.7) (Cantalapiedra et al. 2021) was used to annotate the PSM database file, so Enzyme Commission numbers (EC) terms could be extracted for the analysis. From here, metagenomic reads from our gene catalogues were aligned against this custom database with the blastx algorithm in DIAMOND (v 2.0.15). If identical nucleotide matches were 90% or higher and an *E*‐value of < 10–5 we considered them assigned. To calculate read coverage across potential PSM genes, our methods matched functional profiling. Differences in PSM genes profiles and differential abundance were performed as described above for overall functional profiles.

## 3 Results

### 3.1 Taxonomic Profiling Reveals Rhizosphere Communities Vary Across Milkweed spp. and Are Impacted by Monarch Infestation

**FIGURE 1:**
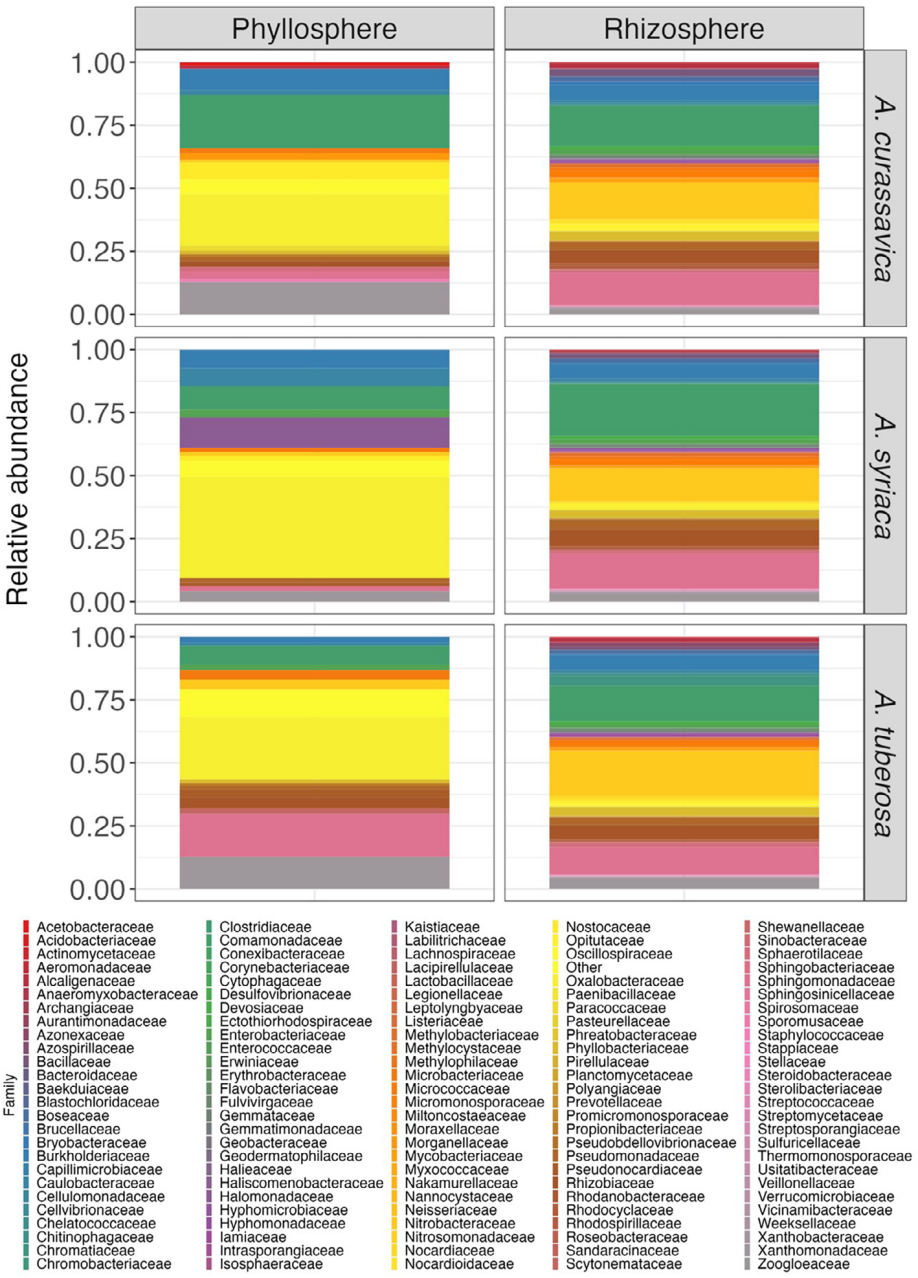
Differences in the taxonomic composition of rhizosphere and phyllosphere associated bacterial communities across milkweed host plant spp. Monarch composition was not taken in account for this figure.

We compared the taxonomic composition of milkweed phyllospheres and rhizospheres to determine if they differed by milkweed spp. and monarch infestation. In total, 585 OTUs were identified, of which two were unique to the phyllosphere, 554 unique to the rhizosphere, and 29 shared between both. Figure 1 summarises bacterial families found in the phyllosphere and rhizosphere. The two most abundant phyllosphere families found across milkweed spp. were Oxalobacteraceae and Xanthomonadaceae. The only other bacterial families above 10% relative abundance were Comamonadaceae and Halomonadaceae, whereas most families were found in low relative abundance. In the rhizosphere, the most abundant bacterial families were shared across all three milkweed spp. (Figure 1). There were additional minor differences in abundances of the families Comamonadaceae (*A. curassavica—*15.9%; *A. syriaca* —20.6%; *A. tuberosa* —14.1%), Nitrobacteraceae (*A. curassavica—*14.6%; *A. syriaca* —12.9%; *A. tuberosa* —17.9%), and Sphingomonadaceae (*A. curassavica—*12.8%; *A. syriaca* —13.9%; *A. tuberosa* —10.8%) (Figure 1). Most of the remaining rhizosphere families were also in relatively low abundance (≤ 1% relative abundance).

**FIGURE 2:**
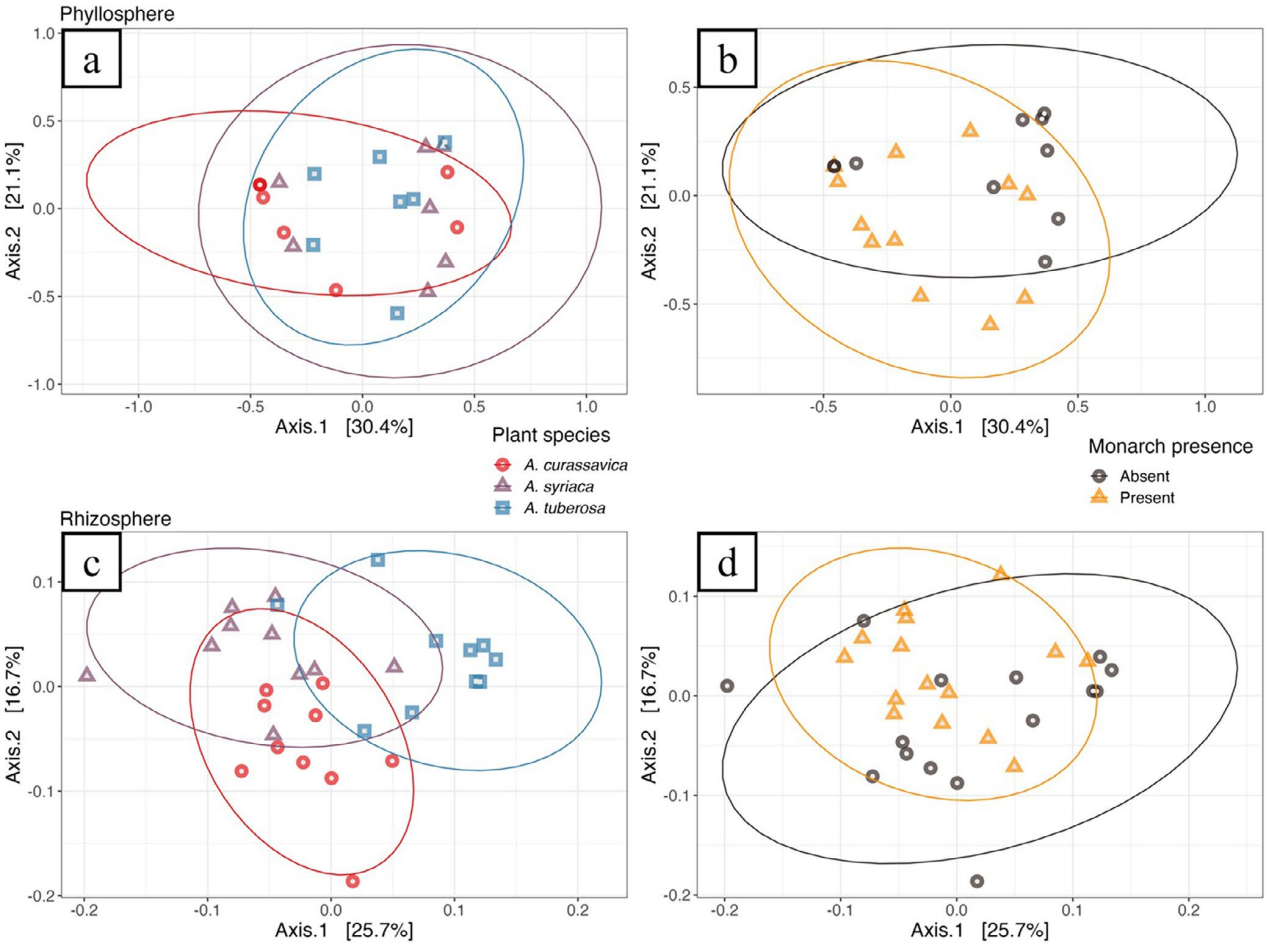
PCoAs of Bray–Curtis dissimilarity showing how the taxonomic profiles of bacterial communities vary in milkweed phyllospheres, by (a) plant spp. and (b) monarch presence, and milkweed rhizospheres, by (c) plant spp. and (d) monarch presence. Results of associated PERMANOVA analyses can be found in Tables S4 and S5.

When we examined alpha diversity of rhizosphere and phyllosphere microbiomes, there were no differences across milkweed spp. (Table S3). Only the number of observed spp. in rhizosphere communities differed between infested and non‐infested plants (Table S3). However, while there were no differences in milkweed phyllosphere community composition across plant spp., rhizosphere communities varied significantly based on Bray–Curtis dissimilarity (Figure 2; Table S4). Plant spp. explained 26.51% of the total variation across rhizosphere samples (Figure 2c). When comparing monarch‐infested plants and un‐infested plants, community differences were significant for rhizospheres (Table S4). Monarch infestation explained 4.72% of the total variation across rhizosphere samples (Figure 2d). The only difference in dispersion found between samples was based on monarch presence in rhizospheres (Table S5). Pairwise comparisons between each plant spp. showed distinct rhizosphere compositions, but similar phyllospheres (Table S6).

### 3.2 Milkweed Phyllospheres and Rhizospheres Have Functionally Unique Sets of Genes Across Plant spp., but Rhizospheres Share a Large Core Set of Genes

**FIGURE 3:**
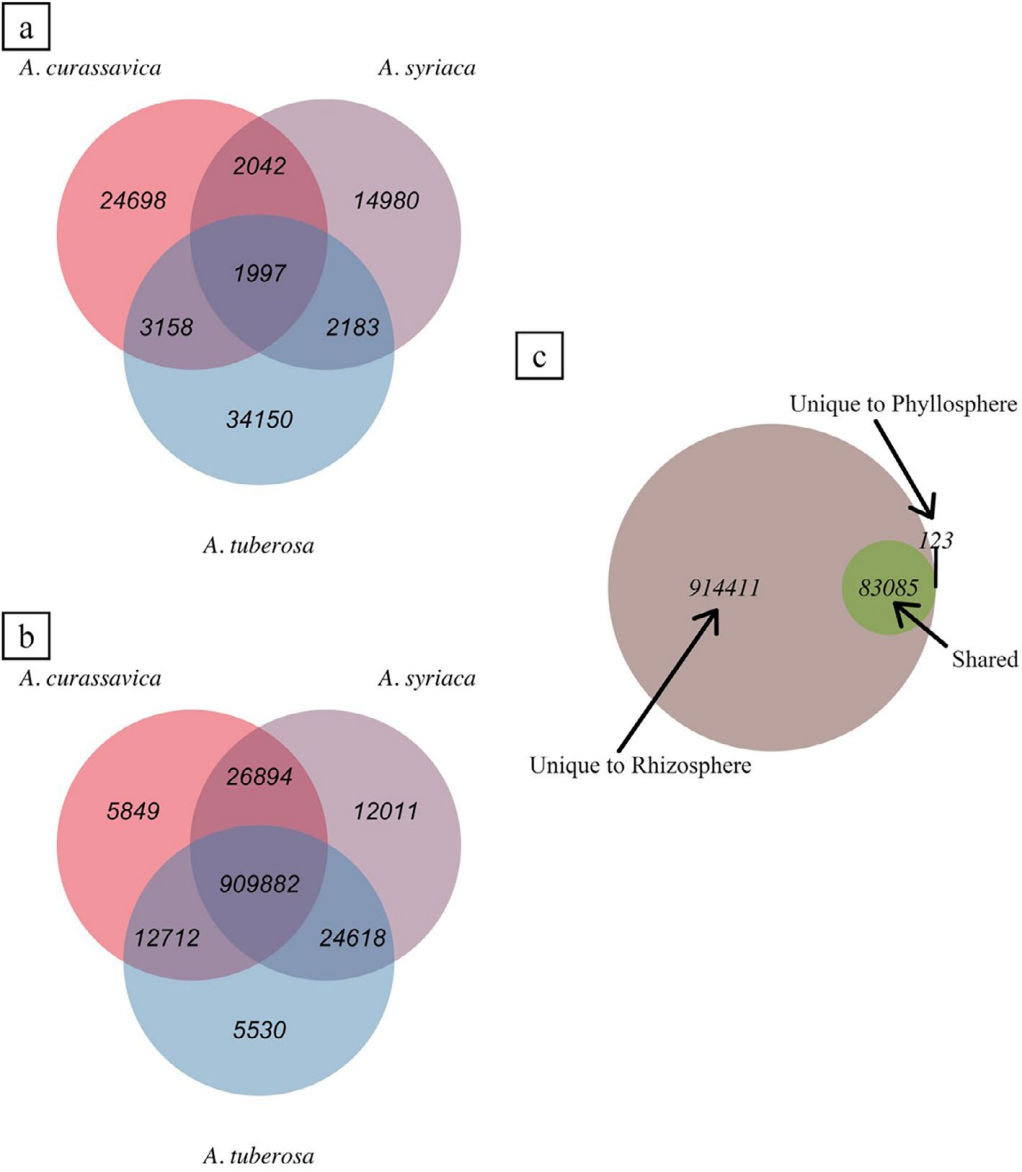
Summary of total number of functionally characterised bacterial genes, with assigned KOs, that were shared or unique across milkweed spp. for (a) the phyllosphere, (b) the rhizosphere, and (c) those shared between phyllosphere and rhizosphere.

In total there were 10,769,385 predicted bacterial genes across both the phyllosphere and rhizosphere, and of those 1,482,817 were annotated. Of these, 83,208 and 997,496 genes had KOs in the phyllosphere and rhizosphere, respectively. Overall, the phyllosphere function profile is distinct between milkweed spp. (Figure 3a). Rhizosphere functional profiles showed the opposite pattern, where a large shared functional core of genes was present (91.22% of genes shared) (Figure 3b). Interestingly, the vast majority of phyllosphere genes identified across all three spp. were shared with the rhizosphere, whereas the rhizosphere had many unique genes (Figure 3c).

**FIGURE 4:**
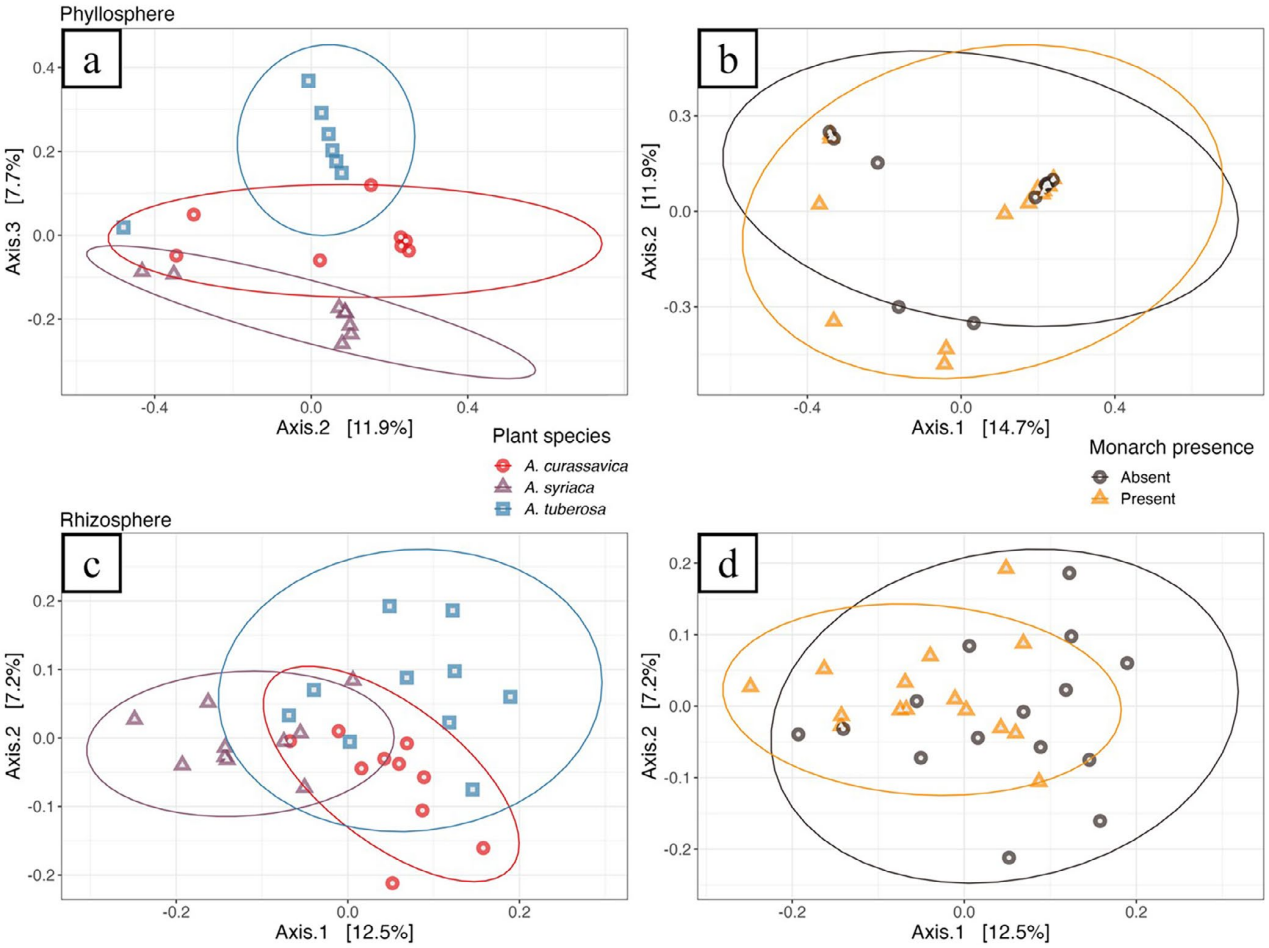
PCoAs of Bray–Curtis dissimilarity showing how the functional profile of bacterial genes with assigned KOs varies in milkweed phyllospheres, by (a) plant spp. and (b) monarch presence, and milkweed rhizospheres, by (c) plant spp. and (d) monarch presence. Results of associated PERMANOVA analyses can be found in Tables S8 and S10.

When genes were grouped by KEGG orthology functional category, there were similar distributions across milkweed spp. For the phyllosphere, the top five functional categories with the most genes found across all three spp. were: ‘protein families: signaling and cellular processes’, ‘Protein families: genetic information processing’, ‘amino acid metabolism’, ‘organismal systems’, and ‘carbohydrate metabolism’ (Table S7). The same pattern was seen in the rhizosphere: ‘protein families: signalling and cellular processes’, ‘carbohydrate metabolism’, ‘Protein families: genetic information processing’, ‘Protein families: metabolism’, and ‘organismal systems’ (Table S7). However, PERMANOVA analysis indicates functional gene profiles were overall distinct between plant spp. for the phyllosphere (*R* ^2^ = 0.21, *p* = 0.0001) and rhizosphere (*R* ^2^ = 0.14, *p* = 0.0001) (Figure 4a,c; Table S8), which was supported by pairwise comparisons between individual milkweed spp. (Table S9). There was some dispersion based on plant spp. in the phyllosphere (Table S10). PERMANOVA analysis also revealed a significant interaction between plant spp. and insect presence in the rhizosphere (*R* ^2^ = 0.084, *p* = 0.0076) (Table S8). We split the data, by samples, by insect treatment and ran individual models for monarch infested and un‐infested plants. Rhizosphere functional gene profiles were overall distinct between plant spp. during monarch absence (*R* ^2^ = 0.25, *p* = 0.0001) and monarch presence (*R* ^2^ = 0.23, *p* = 0.0001) (Table S11). Pairwise comparisons for both monarch absence and monarch presence, further supported these results (Table S12). There were no differences in dispersion between samples for insect treatment (Table S13).

**FIGURE 5:**
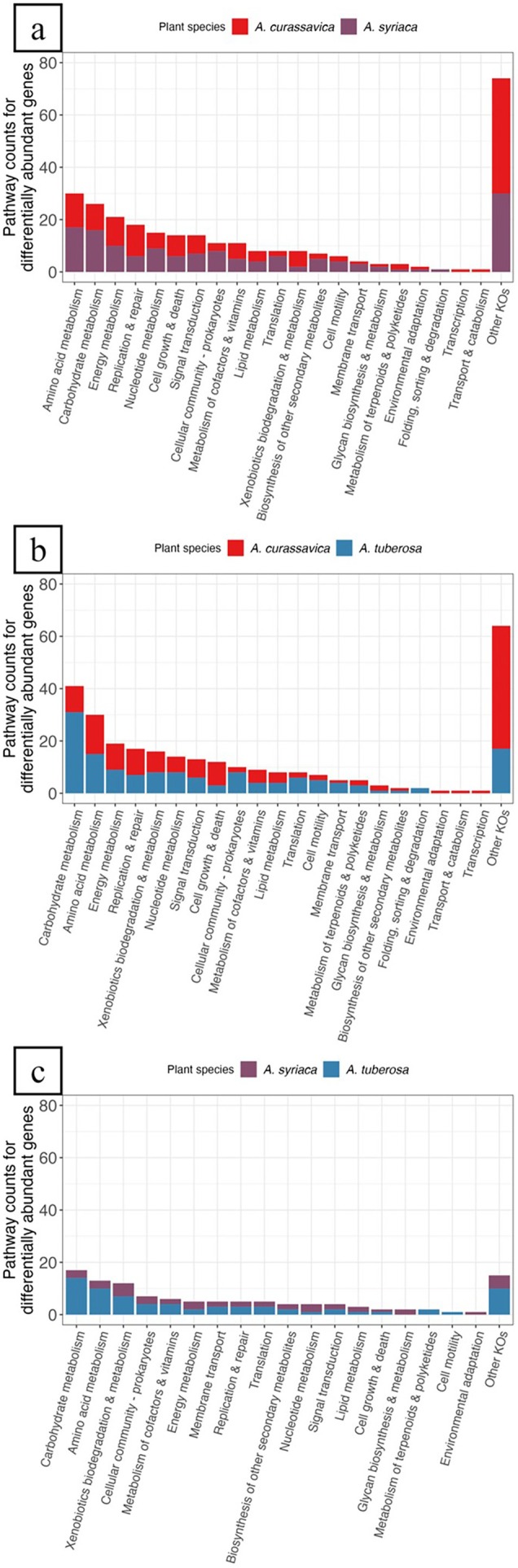
Summary of differential gene abundance analysis in the phyllosphere. Pairwise comparisons between milkweed spp. include (a) *Asclepias curassavica* versus *Asclepias syriaca* , (b) *A. curassavica* versus *Asclepias tuberosa* , and (c) *Asclepias syriaca* versus *Asclepias tuberosa* . Differentially abundant genes that were significantly enriched (i.e., log2 fold change ≥ 2; p‐adjusted ≤ 0.05) are grouped by KEGG functional categories (level two), based on KEGG pathway assignments, and coloured by milkweed spp. The ‘Other KOs’ category consists of genes that were part of KEGG pathways that were not biologically relevant to the phyllosphere or rhizosphere. Results of associated KEGG pathway counts can be found in Tables S14–S16.

We further evaluated differentially abundant genes in the phyllosphere and rhizosphere between each pair of milkweed spp. Overall, there were similar functional categories and pathways represented across all comparisons, but rhizospheres had overall more differentially abundant genes compared to phyllospheres. In the phyllosphere, there were 59 total genes enriched in *A. curassavica* and 88 enriched in *A. syriaca* . These differentially abundant genes mapped to 99 total pathways in *A. curassavica* and 113 total pathways in *A. syriaca* . The top functional categories represented were amino acid metabolism, carbohydrate metabolism, energy metabolism, replication and repair, and nucleotide metabolism (Figure 5a; Table S14). Carbohydrate metabolism had the second most pathways present. Energy metabolism pathways were slightly more represented in *A. curassavica*. Comparing *A. curassavica* — *A. tuberosa* , 56 total genes were enriched in *A. curassavica* and 103 enriched in *A. tuberosa* . These differentially abundant genes mapped to 99 total pathways in *A. curassavica* and 125 total pathways in *A. tuberosa* . The top functional categories represented were carbohydrate metabolism, amino acid metabolism, energy metabolism, replication and repair, and xenobiotics biodegradation and metabolism (Figure 5b; Table S15). Pathways associated with carbohydrate metabolism had slightly higher representation in *A. tuberosa* . Amino acid metabolism was the second largest category. Energy metabolism pathways were slightly more represented in *A. tuberosa* . Comparing *A. syriaca* — *A. tuberosa* , 37 genes were enriched in *A. syriaca* and 48 enriched in *A. tuberosa* . In terms of total pathway counts, there were 38 total pathways in *A. syriaca* and 60 total pathways in *A. tuberosa* . The top functional categories represented were carbohydrate metabolism, amino acid metabolism, xenobiotics biodegradation and metabolism, cellular community—prokaryotes, and metabolism of cofactors and vitamins (Figure 5c; Table S16). Carbohydrate metabolism was more represented in *A. tuberosa* . Amino acid metabolism was also higher in *A. tuberosa* . Cellular community—prokaryotes was more present in *A. tuberosa* .

**FIGURE 6:**
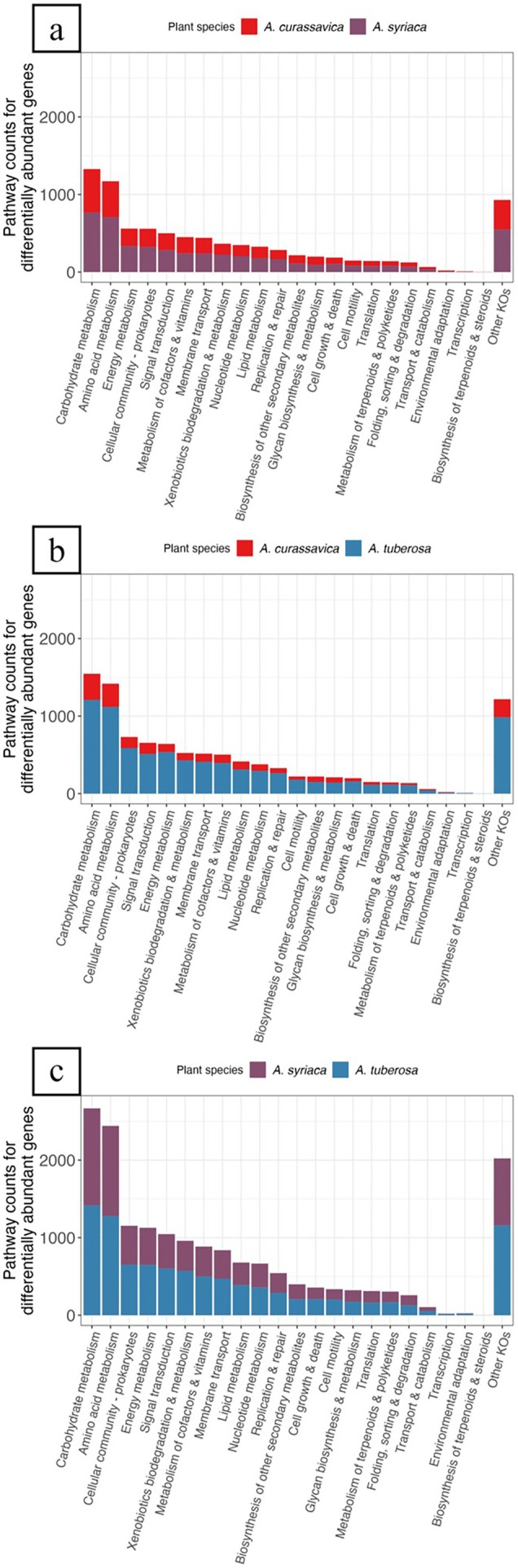
Summary of differential gene abundance analysis in the rhizosphere. Pairwise comparisons between milkweed spp. include (a) *Asclepias curassavica* versus *Asclepias syriaca* , (b) *Asclepias curassavica* versus *Asclepias tuberosa* , and (c) *Asclepias syriaca* versus *Asclepias tuberosa* . Differentially abundant genes that were significantly enriched (i.e., log2 fold change ≥ 2; Padjusted ≤ 0.05) are grouped by KEGG functional categories (level two), based on KEGG pathway assignments, and coloured by milkweed spp. The ‘Other KOs’ category consists of genes that were part of KEGG pathways that were not biologically relevant to the phyllosphere or rhizosphere. Results of associated KEGG pathway counts can be found in Tables S17–S19.

In rhizosphere communities, 2729 total genes were enriched in *A. curassavica* and 3349 enriched in *A. syriaca* . For these genes, there was a difference in total pathway counts of 3241 in *A. curassavica* and 4338 in *A. syriaca* . The top functional categories represented were carbohydrate metabolism, amino acid metabolism, energy metabolism, cellular community—prokaryotes, and signal transduction (Figure 6a; Table S17). Most pathways associated with carbohydrate metabolism, amino acid metabolism, energy metabolism, cellular community—prokaryotes, and signal transduction had a greater presence in *A. syriaca* compared to *A. curassavica* (Table S17). Comparing *A. curassavica* — *A. tuberosa* , 1582 total genes were enriched in *A. curassavica* and 5170 enriched in *A. tuberosa* . When looking at total pathway counts for these genes, 1927 were in *A. curassavica* and 7096 were in *A. tuberosa* . The top functional categories represented were carbohydrate metabolism, amino acid metabolism, cellular community—prokaryotes, signal transduction, and energy metabolism (Figure 6b; Table S18). Again, most pathways associated with carbohydrate metabolism, amino acid metabolism, cellular community—prokaryotes, signal transduction, and energy metabolism had fewer counts in *A. curassavica*. Finally, comparing *A. syriaca* — *A. tuberosa* , there were 5255 total genes enriched in *A. syriaca* and 6370 enriched in *A. tuberosa* . In terms of total pathway counts, there were 6956 pathways in A. syriaca and 8498 pathways in *A. tuberosa* . The top functional categories represented were carbohydrate metabolism, amino acid metabolism, cellular community—prokaryotes, energy metabolism, and signal transduction (Figure 6c; Table S19). Compared to the other plant spp. contrasts, most pathways associated with carbohydrate metabolism, amino acid metabolism, cellular community—prokaryotes, energy metabolism, and signal transduction had similar numbers in both spp. (Table S19).

### 3.3 Monarch Feeding Is Associated With Greater Functional Gene Enrichment in Milkweed Rhizospheres Compared to Phyllospheres

**FIGURE 7:**
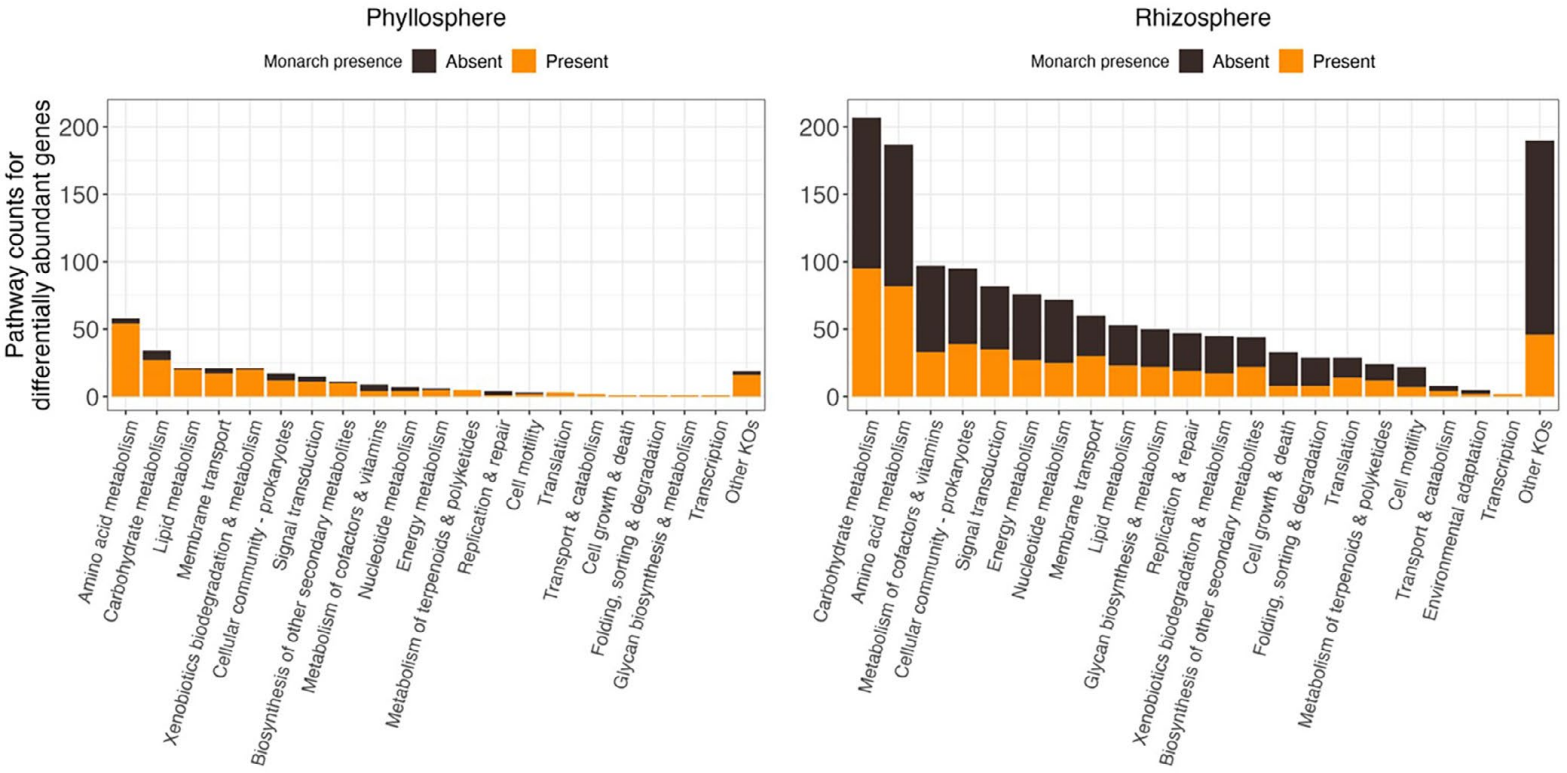
Summary of differential gene abundance analysis in response to monarch infestation in the phyllosphere and rhizosphere. Differentially abundant genes that were significantly enriched (i.e., log2 fold change ≥ 2; p‐adjusted ≤ 0.05) are grouped by KEGG functional categories (level two), based on KEGG pathway assignments, and coloured by monarch presence or absence. The ‘Other KOs’ category consists of genes that were part of KEGG pathways that were not biologically relevant to the phyllosphere or rhizosphere. Associated KEGG pathway counts can be found in Table S22.

Functional gene profiles were overall distinct by monarch presence for the rhizosphere (*R* ^2^ = 0.049, *p* = 0.0057) (Figure 4b,d; Table S8). There was dispersion between samples based on monarch presence (*F* = 6.162, *p* = 0.0181) (Table S10). As stated earlier, PERMANOVA analysis did find a significant interaction between plant spp. and insect presence (Table S8). We split the data by each respective plant spp. and then tested individual models. For the individual models, functional gene profiles were overall distinct by insect presence in the rhizosphere, for *A. curassavica* (*R* ^2^ = 0.17, *p* = 0.0089) and *A. syriaca* (*R* ^2^ = 0.17, *p* = 0.0062) but not *A. tuberosa* (Table S20). There was no dispersion between plant spp. based on insect presence (Table S21). In phyllosphere communities, there are 46 total genes significantly enriched in un‐infested plants and 114 genes enriched in plants fed on by monarchs. The total pathway counts for these genes, in this comparison, were 40 in un‐infested plants and 201 in plants fed on by monarchs. The top functional categories represented were amino acid metabolism, carbohydrate metabolism, lipid metabolism, membrane transport, and xenobiotics biodegradation and metabolism (Figure 7; Table S22). Most pathways found under these categories' either had greater total counts or were unique to monarch‐infested phyllospheres.

Monarch feeding had less impact on rhizosphere communities, as there were 647 total genes enriched in monarch absence compared to 442 genes enriched in monarch presence. The total pathway counts for these genes, in this comparison, were 741 in un‐infested plants and 526 in plants fed on by monarchs. The top functional categories represented were carbohydrate metabolism, amino acid metabolism, metabolism of cofactors and vitamins, cellular community—prokaryotes, and signal transduction (Figure 7; Table S23). Many pathways had similar counts in monarch‐infested versus un‐infested plants.

### 3.4 Phyllospheres and Rhizospheres Have Distinct PSM Gene Compositions That Differ Across Milkweed spp., but Are Not Impacted by Monarch Feeding

From broad functional profiling of bacterial genes, there were two categories identified with differentially abundant genes: metabolism of terpenoids and polyketide, and xenobiotics biodegradation and metabolism. For the phyllosphere, the largest PSM pathway counts were associated with *A. curassavica* and *A. tuberosa* (Table S24). Many were single counts. Interestingly, for infested versus un‐infested, the majority of PSM metabolism counts were for infested phyllospheres, with the Benzoate degradation pathway as the most prevalent. The only significantly enriched pathways within the phyllosphere (Atrazine degradation, Benzoate degradation, and Pinene, camphor, and geraniol degradation) were all associated with monarch infestation (Table S25).

Within the rhizosphere, total pathway counts of PSM metabolism associated pathways were greatest in *A. tuberosa* , followed by *A. syriaca* and *A. curassavica* (Table S26). This included the pathways of aminobenzoate degradation, benzoate degradation, biosynthesis of ansamycins, caprolactam degradation, chloroalkane and chloroalkene degradation, chlorocyclohexane and chlorobenzene degradation, and limonene degradation, which were all substantially greater and followed the same plant species trend. Many of the same pathways found with differential abundance were also significantly enriched across plant species comparison treatments (Table S25). Overwhelmingly, most of the enriched pathways were seen in *A. tuberosa* rhizospheres. When comparing infested and uninfested plants, there were similar numbers of total pathways across functional categories linked to potential PSM metabolism. There were only two enriched pathways related to uninfested plants, indicating monarch infestation may have little influence on PSM metabolism in plant rhizospheres.

**FIGURE 8:**
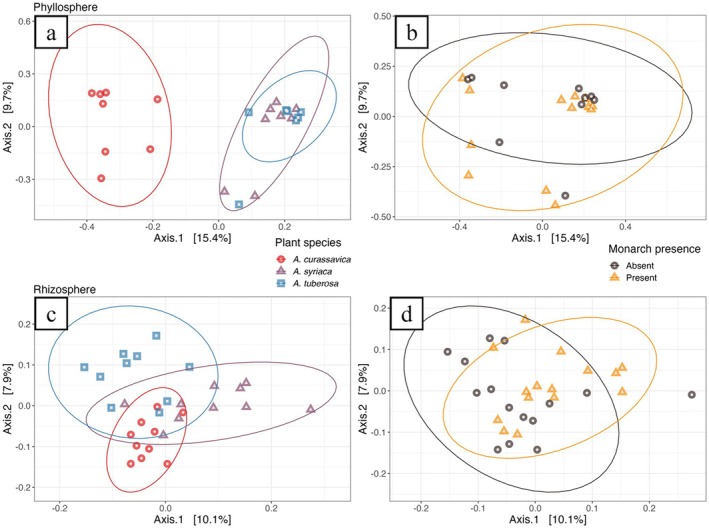
PCoAs of Bray–Curtis dissimilarity showing how potential microbial PSM metabolism gene profiles change in milkweed phyllospheres, by (a) plant spp. and (b) monarch presence, and milkweed rhizospheres, by (c) plant spp. and (d) monarch presence. Results of associated PERMANOVA analyses can be found in Tables S27 and S29.

In addition, we performed a targeted analysis using a custom built PSM gene/enzyme database. Our database was composed of c. 1,152,814 total genes. We then filtered aligned hits, leaving us with 42,184 potential candidates. The PSM gene profiles for both phyllosphere and rhizosphere communities were distinct across milkweed plant spp., explaining 21% and 12% of overall variation (Figure 8a,c; Tables S27 and S28), which were further confirmed by pairwise comparisons (Table S28). However, PSM gene profiles in phyllospheres and rhizospheres did not differ by insect treatment (Figure 8b,d; Table S27). Only the dispersion effect was in the rhizosphere with monarch presence (*F* = 4.6643, *p* = 0.0249) (Table S29).

Next, we performed differential gene abundance analysis between all pairs of milkweed spp. Overall, in the phyllosphere, there were only 10 total differentially abundant PSM genes between each of the comparisons. In contrast, the rhizosphere had 306 total differentially abundant PSM genes between each of the comparisons. For each of these comparisons, we then connected EC information back to the differentially abundant PSM genes (for genes with ECs) (Table S30). Among phyllosphere communities, three enzymes were greater in *A. curassavica* compared to the other spp.: beta‐N‐acetylhexosaminidase, betaporphyranase, exo‐alpha‐sialidase. Only one enzyme was greater in *A. syriaca—*beta‐galactosidase. Finally, cytochrome‐c oxidase was greater in *A. tuberosa* compared to the other spp. In rhizosphere communities, we found overall fewer total PSM enzymes in *A. curassavica* compared to the other two spp. For example, *A. syriaca* and *A. tuberosa* have similar total numbers of cytochrome‐c oxidases, whereas these enzymes were in lower abundance or absent in *A. curassavica*.

## 4 Discussion

We investigated how milkweed‐associated microbiomes functionally differ across milkweed spp. known to vary in defensive chemical profiles. To do so, we sequenced milkweed endophytic phyllosphere and rhizosphere bacterial communities, then identified functional gene profiles across different milkweed spp. that were either exposed to monarch feeding or uninfested. Overall, we found endophytic phyllosphere microbes had sets of functional genes distinct to each milkweed spp., whereas rhizosphere microbes shared a core set of functional genes. However, functional gene profiles for both the phyllosphere and rhizosphere were generally distinct between plant spp. and varied in abundance of genes predicted to metabolise PSMs through various pathways. Interestingly, monarch infestation caused significant shifts in the overall composition of functional gene profiles for the rhizosphere, but did not specifically impact the presence of PSM associated genes or pathways.

Variation in functional gene profiles between endophytic phyllosphere and rhizosphere is likely linked to differences in environmental conditions experienced by these microbial communities. The endophytic microbiota of the phyllosphere is less affected by external abiotic conditions (e.g., UV radiation, humidity) and instead is thought to exist in a more stable internal environment with greater available nutrients (Dastogeer et al. 2020; Sohrabi et al. 2023). However, endophytes are more directly influenced by differences in plant physiology linked to genotype and immune responses (Chaudhry et al. 2021; Dastogeer et al. 2020; Sohrabi et al. 2023; Xu et al. 2022). Although the rhizosphere environment can also be stable to some degree and nutrient rich, competition between a greater diversity of microbes is a major factor shaping these communities (Park et al. 2023; Singh et al. 2023). Functional potential of the phyllosphere and rhizosphere could also be impacted by different PSM levels, particularly cardenolides. Endophytic phyllosphere communities are likely more directly influenced by PSMs (Sohrabi et al. 2023) compared to rhizosphere microbes. For example, milkweed spp. vary in storage and amounts of latex (which has high cardenolide concentrations) found in leaves (Agrawal 2004; Agrawal et al. 2014; Agrawal and Fishbein 2006). However, cardenolides have greater concentration and diversity in root tissues compared to shoot tissues (Rasmann and Agrawal 2011). PSMs exuded through roots (Korenblum et al. 2020) as well as unique exudates used for recruitment of specific microbes (Jain et al. 2020; Qu et al. 2020; Vives‐Peris et al. 2020) could also contribute to differences in functional potential. However, it is currently unclear to what extent cardenolides and PSMs are being exuded by milkweed roots.

Few studies have investigated milkweed associated microbiota at the community level (Hansen and Enders 2022; Warren et al. 2020). Our previous work (Hansen and Enders 2022) characterising milkweed rhizosphere and phyllosphere microbiomes, and the current study are therefore important for establishing baseline information on taxonomic profiles. Overall, Hansen and Enders (2022) found 56 total bacterial families compared to 130 total families in the current study. However, milkweed rhizospheres were generally similar, with several dominant taxa present in both studies (i.e., Burkholderiaceae, Nostocaceae, Pseudomonadaceae, Rhizobiaceae, Sphingomonadaceae, Xanthobacteraceae) and variation in community composition observed across plant spp. There were fewer similarities for phyllosphere communities across studies. Hansen and Enders (2022) found community composition varied across plant spp., whereas monarch infestation did not have an impact. In contrast, our results here showed phyllosphere community composition did not vary across plant spp., but monarch infestation did have an impact. Some variation in taxonomic profiles is to be expected, given differences in experiments conducted (e.g., number of plant spp., sequencing technology).

Finally, it is important to note that taxonomic profiling was performed at the family level in the current study, which could partially explain why phyllosphere functional profiles differ among milkweed species without significant taxonomic variation. Individual species and strains within a bacterial family are predicted to vary in genomic content, which could translate to differences in functional gene profiles at the genus or species level. Alternatively, horizontal gene transfer between taxa (Ramond et al. 2024) and host plant selection for different taxa could be altering gene content within bacterial families found across milkweed spp. In contrast, our results showed significant differences in rhizosphere taxonomic composition but the absence of variation in functional profiling, which suggests recruitment of different taxa with functional redundancy from bacterial families with similar metabolic roles for example distinct taxa have redundancy in polysaccharide‐degrading capacity (Li et al. 2024) Functional redundancy is a documented phenomenon in soil microbial communities that can be observed at a global scale (Chen, Ma, et al. 2022).

### 4.1 Functional Profiles of Milkweed Microbiomes Suggest Potential for PSM Metabolism

We hypothesised defensive chemical profiles impose positive directional selection on plant‐associated microbiomes, causing an enrichment of detoxification genes and pathways across bacterial genomes. Using a targeted gene approach, we focused on characterising evidence of PSM metabolism in bacterial metagenomes associated with cardenolides, alkaloids, flavonoids, phenolics, saponins, tannins, and terpenoids. We further predicted enrichment of PSM metabolism genes is greatest in metagenomes of milkweed spp. with higher expression/concentrations of cardenolides (i.e., tropical, and common milkweed). Using our custom database of genes predicted to metabolise PSMs, we found the composition of potential PSM genes varied across milkweed spp. with differences between rhizospheres and phyllospheres.

Glycoside hydrolases and oxidoreductases/translocases are two candidate enzyme families with potential to break down cardenolides and other PSMs. Glycoside hydrolases are a wide family of enzymes that cleave carbohydrate/sugars (Drula et al. 2022). It is possible many of these glycoside hydrolases have a function against PSMs, as seen with ginsenoside metabolism in *Terrimonas ginsenosidimutans* sp. nov. (Siddiqi et al. 2023). Oxidoreductases/translocases could be involved in cardenolide reduction, as suggested by previously identified digoxin reductase proteins (Cgr1 and Cgr2) (Koppel et al. 2018). Several glycoside hydrolase genes were identified in both the phyllosphere and rhizosphere. For example, alpha‐L‐fucosidase and beta‐galactosidase might be candidates for cardiac glycoside degradation. In western honeybees ( *Apis mellifera* ) these enzymes helped to degrade the PSM amygdalin by hydrolyzing the sugar group (Motta et al. 2022). Previous research with other plant spp. has shown the enzyme beta‐glucosidase, found associated with *A. curassavica* in this study, can deglycosylate cardenolides (Eisenbeiß et al. 1999; Knittel et al. 2015; Schöniger et al. 1998). The presence of several oxidoreductase indicates another possible route of cardenolide metabolism. Across all three rhizospheres, we found numerous cytochrome oxidoreductase enzymes, with the highest count for cytochrome‐c oxidase explicitly. It is important to mention glycoside hydrolases and cytochrome oxidoreductases could be involved with generalised functions not associated with PSM metabolism. Glycoside hydrolases could be involved in the metabolism of a range of rhizosphere exudates, as seen with alpha‐L‐fucosidase, beta‐agarase, beta‐galactosidase, and glucan 1,4‐alphaglucosidase. Cytochrome oxidoreductases could be involved with reactive oxygen species stress responses (Mandon et al. 2021; Qi et al. 2020; Yang et al. 2021) or microbial respiration (Abramson et al. 2000; Kelly et al. 1990; Schimo et al. 2017; Smith et al. 1990).

We found evidence for microbial PSM metabolism pathways (partial and complete) across both the phyllosphere and rhizosphere, for alkaloids, flavonoids, phenolics, saponins, tannins, and terpenoids. Specifically, we found partial pathways for nicotine and caffeine degradation, which can be used by bacteria for alkaloid degradation (Mu et al. 2020; Vega et al. 2021). Partial flavonoid degradation was present, but differential abundance of specific genes was not detected. We also found partial pathways for limonene degradation and pinene, camphor and geraniol degradation, which could be linked to terpenoid metabolism (Förster‐Fromme et al. 2006; Griffiths et al. 1987; Höschle et al. 2005; Van Der Werf et al. 1999; Van Der Werf and Boot 2000). The capability for milkweed microbes degrading saponin is also likely, as we found a complete pathway for steroid degradation: which, in combination with glucoside hydrolyses, can lead to saponin degradation (Li et al. 2022; Nakayasu et al. 2023; Zhang et al. 2022). Steroid degradation also had high pathway counts for our rhizosphere samples. Finally, there is potential for phenolic degradation, as complete pathways for benzoate degradation, benzoyl‐CoA degradation, catechol meta‐cleavage, catechol ortho‐cleavage were detected (Chen, Hu, et al. 2022; Harzallah et al. 2023; Luo et al. 2023; Van Dexter and Boopathy 2019; Yu et al. 2023; Zhang et al. 2022, 2023). Finally, pathways directly related to degradation of aromatic compounds: aminobenzoate degradation, benzoate degradation, naphthalene degradation, polycyclic aromatic hydrocarbon degradation, styrene degradation, toluene degradation, and xylene degradation suggest capacity for carbon utilisation and bioremediation of xenobiotic pollutants (Asemoloye et al. 2019; Kuiper et al. 2004; Musilova et al. 2016; Rohrbacher and St‐Arnaud 2016; Singha and Pandey 2021; Vasudevan et al. 2018; Waigi et al. 2017; Yang et al. 2020).

We predicted that the enrichment of PSM metabolism genes is greatest in metagenomes of plants exposed to monarch caterpillar feeding compared to un‐infested plants. Overall, we did not find strong evidence that insect herbivore feeding causes changes in the potential for microbial PSM metabolism in milkweed microbiomes, although the broader functional gene profiles of the rhizosphere showed minor changes in response to monarch feeding for two of our milkweed species ( *A. curassavica* and *A. syriaca* ). Previous research has shown that insect herbivore feeding can impact microbial communities in the phyllosphere (Humphrey and Whiteman 2020; Potthast et al. 2022) and rhizosphere (Friman et al. 2021; Ourry et al. 2018). However, it is possible that the duration of monarch feeding used in this experiment was not long enough to cause the enrichment of PSM metabolism genes in milkweed microbiomes. Cardenolide induction in response to herbivory varies by milkweed spp. and genotype, and non‐linear induction patterns have been documented (Agrawal et al. 2012). *A. curassavica* and *A. syriaca* also show differences in the induction and production of cardenolides depending on levels of arbuscular mycorrhizal fungi (AMF) colonisation (Meier and Hunter 2018; Vannette et al. 2013). Additional work is needed to investigate the effects of longer monarch feeding across a range of milkweed genotypes to fully understand impacts on rhizosphere and phyllosphere microbiomes. Our work suggests that differences in the functional roles of rhizosphere microbes could also be important in mediating the expression of plant defences against insects. A large body of research shows that root microbes can induce systemic defences against insect attackers (Pineda et al. 2010; Rashid and Chung 2017) and promote greater tolerance to damage (Tronson and Enders 2025), but it remains unclear how specific microbial functions are connected to the expression of defences and ultimately variation in insect resistance.

## 5 Conclusion

Our study found evidence of microbial PSM metabolism in the phyllospheres and rhizospheres of three milkweed spp. ( *A. curassavica* , *A. syriaca* , and *A. tuberosa* ). Contrary to predictions, the abundance of pathways and enzymes connected to PSM metabolism showed an inverse pattern in relation to the milkweed defensive chemical profile (i.e., greatest in *A. tuberosa* and lowest in *A. curassavica* ). Results also suggest a greater potential for PSM detoxification in the rhizosphere compared to endophytic phyllosphere communities. Although interesting, these results indicate that more research needs to be done to elucidate the complex interactions between milkweeds, monarchs, and microbes. Further work should focus on investigating signatures of selection across PSM genes and microbial genomes. Measuring cardenolide exudation patterns in roots and shoots, in tandem with microbial taxonomic profiling, could uncover specific interactions between cardenolides and microbiome assembly. Metabolomic profiling of roots and leaves could identify other PSMs important in shaping milkweed microbial communities and the potential for microbial‐derived PSMs.

## Author Contributions

**Thorsten E. Hansen:** conceptualization, data curation, formal analysis, investigation, methodology, project administration, software, visualization, writing – original draft, writing – review and editing. **Laramy S. Enders:** conceptualization, funding acquisition, methodology, project administration, resources, supervision, writing – review and editing.

## Conflicts of Interest

The authors declare no conflicts of interest.

## Supporting information


**Table S1.** Global Positioning System (GPS) coordinates of the site soil was sampled from in Indiana, United States of America.
**Table S2.** Sequencing summary. A list of each individual sample sequenced, the different treatments that sample was a part of, the initial number of reads samples had after base quality control, and the number of reads after removal of any host contamination (human, milkweed, and monarch).
**Table S3.** Summary of results from Kruskal‐Wallis tests on alpha diversity metrics compared across plant spp. and insect presence in the phyllosphere and rhizosphere.
**Table S4.** Summary of PERMANOVA of Bray–Curtis dissimilarity comparing taxonomic profiles of phyllosphere and rhizosphere communities across plant spp. and insect presence.
**Table S5.** Summary of PERMDISP test of Bray–Curtis dissimilarity for taxonomic profiles of phyllosphere and rhizosphere communities across plant spp. and insect presence.
**Table S6.** Summary of pairwise PERMANOVA of Bray–Curtis dissimilarity for each microbiome type (phyllosphere and rhizosphere). These comparisons tested for differences in taxonomic composition between each individual plant spp.
**Table S7.** Summary of total gene relative abundance (for genes with assigned KOs), grouped by KEGG orthology functional category, for both the phyllosphere and rhizosphere in each plant spp.
**Table S8.** Summary of PERMANOVA of Bray–Curtis dissimilarity for functional profiling of bacterial genes for each microbiome type (phyllosphere and rhizosphere) comparing across plant spp. and insect presence.
**Table S9.** Summary of pairwise PERMANOVA of Bray–Curtis dissimilarity (for functional profiling of bacterial genes) for each microbiome type (phyllosphere and rhizosphere). These comparisons tested for differences in composition between functional gene profiles between each individual plant spp.
**Table S10.** Summary of PERMDISP test of Bray–Curtis dissimilarity (for functional profiling of bacterial genes) comparing plant spp. for each microbiome type (phyllosphere and rhizosphere) and insect presence for milkweed microbiomes.
**Table S11.** Summary of individual PERMANOVA models, separately testing the plant spp. comparison for all samples that have monarchs and those that do not, for the rhizosphere. Each model used Bray–Curtis dissimilarity to test the differences in composition between functional profiles of bacterial genes with assigned KOs.
**Table S12.** Summary of individual pairwise PERMANOVA of Bray–Curtis dissimilarity (separately testing the plant spp. comparison for all samples that have monarchs and those that do not) for the rhizosphere. These comparisons tested for differences in composition between functional gene profiles between each individual plant spp.
**Table S13.** Summary of PERMDISP test of Bray–Curtis dissimilarity of individual PERMANOVA models (separately testing the plant spp. comparison for all samples that have monarchs and those that do not) for the rhizosphere.
**Table S14.** Summary of KEGG pathway counts, for the top five KEGG functional categories, for differentially abundant genes in the phyllosphere *Asclepias curassavica* versus *Asclepias syriaca* comparison.
**Table S15.** Summary of KEGG pathway counts, for the top five KEGG functional categories, for differentially abundant genes in the phyllosphere *Asclepias curassavica* versus *Asclepias tuberosa* comparison.
**Table S16.** Summary of KEGG pathway counts, for the top five KEGG functional categories, for differentially abundant genes in the phyllosphere *Asclepias syriaca* versus *Asclepias tuberosa* comparison.
**Table S17.** Summary of KEGG pathway counts, for the top five KEGG functional categories, for differentially abundant genes in the rhizosphere *Asclepias curassavica* versus *Asclepias syriaca* comparison.
**Table S18.** Summary of KEGG pathway counts, for the top five KEGG functional categories, for differentially abundant genes in the rhizosphere *A. curassavica* versus *A. tuberosa* comparison.
**Table S19.** Summary of KEGG pathway counts, for the top five KEGG functional categories, for differentially abundant genes in the rhizosphere *A. syriaca* versus *A. tuberosa* comparison.
**Table S20.** Summary of individual PERMANOVA models, testing the insect presence comparison for each individual plant spp., for the rhizosphere. Each model used Bray–Curtis dissimilarity to test the differences in composition between functional profiles of bacterial genes with assigned KOs.
**Table S21.** Summary of PERMDISP test of Bray–Curtis dissimilarity of individual PERMANOVA models (testing the insect presence comparison for each individual plant spp.) for the rhizosphere.
**Table S22.** Summary of KEGG pathway counts, for the top five KEGG functional categories, for differentially abundant genes in the phyllosphere for monarch absence versus monarch presence comparison.
**Table S23.** Summary of KEGG pathway counts, for the top five KEGG functional categories, for differentially abundant genes in the rhizosphere for monarch absence versus monarch presence comparison.
**Table S24.** Summary of KEGG pathway counts (for metabolism of terpenoids and polyketides along with xenobiotics biodegradation and metabolism), for differentially abundant genes in the phyllosphere, for each comparison.
**Table S25.** Over representation analysis of differentially abundant genes, by more than 2‐fold, between treatment comparisons based on KEGG pathways (for metabolism of terpenoids and polyketides along with xenobiotics biodegradation and metabolism).
**Table S26.** Summary of KEGG pathway counts (for metabolism of terpenoids and polyketides along with xenobiotics biodegradation and metabolism), for differentially abundant genes in the rhizosphere, for each comparison.
**Table S27.** Summary of PERMANOVA of Bray–Curtis dissimilarity (for PSM database gene alignments) comparing plant spp. for each microbiome type (phyllosphere and rhizosphere) and insect presence for milkweed microbiomes.
**Table S28.** Summary of pairwise PERMANOVA of Bray–Curtis dissimilarity (for PSM database gene alignments) for each microbiome type (phyllosphere and rhizosphere). These comparisons tested for differences in composition between the genes aligned to PSM database for each individual plant spp. comparison.
**Table S29.** Summary of PERMDISP test of Bray–Curtis dissimilarity (for PSM database gene alignments) comparing plant spp. for each microbiome type (phyllosphere and rhizosphere) and insect presence for milkweed microbiomes.
**Table S30.** Summary of EC enzyme pathway counts, for differentially abundant PSM genes, in the phyllosphere and rhizosphere, for each plant spp. comparison.

## Data Availability

The data that support the findings of this study are openly available in the Sequence Read Archive at https://www.ncbi.nlm.nih.gov/bioproject/PRJNA1121629, reference number PRJNA1121629. The tentative release date for the data is 2024‐12‐31. We do intend to make the data publicly available when the manuscript is published.
